# Neutrophil extracellular traps mediated by platelet microvesicles promote thrombosis and brain injury in acute ischemic stroke

**DOI:** 10.1186/s12964-023-01379-8

**Published:** 2024-01-17

**Authors:** Xin Gao, Xinyi Zhao, Jiacheng Li, Chang Liu, Wenqiang Li, Junjie Zhao, Zhixi Li, Nan Wang, Fang Wang, Jiawei Dong, Xiuwei Yan, Jiheng Zhang, Xueyan Hu, Jiaqi Jin, Ge Mang, Ruishuang Ma, Shaoshan Hu

**Affiliations:** 1 Department of Neurosurgery, Cancer Center, Zhejiang Provincial People’s Hospital, Hangzhou Medical College, Hangzhou, Zhejiang China; 2 Department of Neurosurgery, The Second Affiliated Hospital of Harbin Medical University, Harbin, China; 3 Department of Cardiology, The Second Affiliated Hospital of Harbin Medical University, Harbin, China; 4 The Key Laboratory of Myocardial Ischemia, Ministry of Education, Harbin, Heilongjiang Province China; 5 Department of Vascular Surgery, Jinshan Hospital of Fudan University, Shanghai, China; 6 Department of General Surgery, Changsha Fourth Hospital, Changsha, China; 7 Department of Neurosurgery, Xuanwu Hospital, Capital Medical University, Beijing, China; 8 Department of Radiotherapy and Chemotherapy, The First Affiliated Hospital of Ningbo University, Ningbo, China; 9 Department of Radiotherapy and Chemotherapy, Ningbo First Hospital of Ningbo, Ningbo, 315000 China

**Keywords:** Neutrophil extracellular traps, HMGB1, Platelet, Microvesicles, Thrombosis, Stroke

## Abstract

**Aims:**

Neutrophil extracellular traps (NETs) have been implicated in thrombotic diseases. There is no definitive explanation for how NETs form during acute ischemic strokes (AIS). The purpose of our study was to investigate the potential mechanism and role of NETs formation in the AIS process.

**Methods:**

As well as 45 healthy subjects, 45 patients with AIS had ELISA tests performed to detect NET markers. Expression of high-mobility group box 1 (HMGB1) on platelet microvesicles (PMVs) was analyzed by flow cytometry in healthy subjects and AIS patients’ blood samples. We established middle cerebral artery occlusion (MCAO) mice model to elucidate the interaction between PMPs and NETs.

**Results:**

A significant elevation in NET markers was found in patient plasma in AIS patients, and neutrophils generated more NETs from patients’ neutrophils. HMGB1 expression was upregulated on PMVs from AIS patients and induced NET formation. NETs enhanced Procoagulant activity (PCA) through tissue factor and via platelet activation. Targeting lactadherin in genetical and in pharmacology could regulate the formation of NETs in MCAO model.

**Conclusions:**

NETs mediated by PMVs derived HMGB1 exacerbate thrombosis and brain injury in AIS.

Video Abstract

**Supplementary Information:**

The online version contains supplementary material available at 10.1186/s12964-023-01379-8.

## Introduction

Ischemic stroke is a prominent contributor to mortality and disability, constituting 70–90% of cerebral stroke instances and impacting 24.9 million cases globally [1–3]. The treatment of ischemic stroke has been authorized through two primary methods: (1) pharmacological thrombolysis utilizing tissue plasminogen activator (t-PA) and (2) mechanical extraction of the thrombus via endovascular thrombectomy. The limited timeframe available may impede the effectiveness of thrombolytic therapies, and endovascular thrombectomy may not always be a viable alternative. The use of t-PA results in recanalization in fewer than half of those who receive it. Therefore, exploring new therapeutic targets for AIS is urgently needed.

As a component of the innate immune system and a regulator of the adaptive immune system, neutrophils play a key role. Moreover, Neutrophils are the first leukocytes to infiltrate brain ischemia after cerebral infarctions and are considered markers of inflammation [4]. In the acute phase following stroke, neutrophils adhere to endothelial ischemic brain vasculature and infiltrate into brain parenchyma [4–8]. Experiments on stroke models using neutrophil depletion, neutrophil adhesion modulation, or inhibitors of proteolytic enzymes, such as elastase released by neutrophils improve injury size and neurological deficits [9, 10]. In recent decades, neutrophils have been identified as critical players in interaction between thrombosis and inflammation in ischemic stroke through regulating neutrophils extracellular traps (NETs) formation [11–14]. NETs are web-like structures composed of decondensed chromatin coated with granule proteins, such as histones, myeloperoxidase (MPO), and neutrophil elastase (NE) [15, 16]. NETs have been identified as crucial mediators of the coagulation state in thrombotic diseases [17–19]. Researchers have recently shown that NETs contribute significantly to ischemic stroke thrombi, making them a potential therapeutic target [11–13]. However, relatively little is known about the exact mechanism of NET formation in AIS. Crosstalk between activated platelets and neutrophils contributed to NET generation in thrombotic disease [18, 20–22]. When platelets are activated, microvesicles (MPs) derived from the plasma membrane (70–90% of it) are generated [23]. PMPs have been reported as biomarker in stroke patients [24–26]. Nevertheless, the definite crosstalk between PMPs and NETs formation in AIS has been not well studied.

In this study, we assessed NET levels in AIS patients and evaluated the potential role of NETs. We also investigated interaction between NETs and PMPs in the process of AIS. As a result of our novel findings, new therapeutic targets may be identified for the prevention and treatment of vascular complications associated with AIS.

## Materials and methods

### Patients

In this study, we selected 45 control subjects and 45 AIS patients who were admitted to the Second Affiliated Hospital of Harbin Medical University from March 2021 to November 2021. AIS was diagnosed based on definite diagnostic criteria and CT/MRI. AIS thrombi were obtained from patients who underwent thrombectomy. The exclusion criteria were infection, malignancy, liver or renal failure or pregnancy. Study population is showed in Table 1. This study was approved by the Ethics Committee of The Second Affiliated Hospital of Harbin Medical University and was conducted following the specifications of the Declaration of Helsinki.

**Table 1:** Characteristics of the study population

Characteristics	Control (*n* = 45)	AIS (*n* = 45)
Age (years)	58.46 ± 10.4	57.48 ± 11.4
Male (n, %)	(75%)	(75%)
WBC (×10^9^)	6.87 ± 1.32	10.05 ± 3.38*
Neutrophils (%)	59.90 ± 6.57	75.2 ± 10.67*
Monocytes (%)	3.42 ± 0.55	2.83 ± 0.92*
Lymphocytes (%)	21.04 ± 7.25	20.8 ± 9.63*
Eosinophils (%)	1.19 ± 0.67	1.2 ± 1.19
Basophils (%)	0	0
Total cholesterol (mmol/l)	3.16 ± 0.52	4.37 ± 0.78
Triglycerides (mmol/l)	1.42 ± 0.41	1.52 ± 1.21
LDL (mmol/l)	1.58 ± 0.36	2.88 ± 0.76*
HDL (mmol/l)	0.82 ± 0.20	1.16 ± 0.29
NIHSS score	—	8.69 ± 5.67
Previous AIS or TIA (n)	—	5
Diabetes (n)	2	6
Hypertension (n)	—	11
Hypercholesterolemia (n)	0	12
Cigarette smoking (n)	0	14
Erythrocytes(×10^12^/l)	4.33 ± 0.35	4.74 ± 0.46
PLT (×10^9^)	259.2 ± 62.29	221.52 ± 47.6*
PT (s)	11.24 ± 1.04	11.03 ± 0.78
APTT (s)	35.06 ± 2.70	31.99 ± 3.52
D-dimer (mg/l)	41.8 ± 25.4	263.07 ± 208.6*
Fibrinogen (mg/l)	2.46 ± 0.43	3.07 ± 0.52*
Pharmacological therapy		
Antiplatelet (n)	—	20
Anticoagulant (n)	—	5
Fibrinolysis (n)	—	25
ACE inhibitor or ARB (n)	6	18
Statin (n)	4	22

The main clinical and laboratory features of 45 healthy controls and 45 patients diagnosed with stroke. Data are presented as numbers (percentages) or the median ± SD

*WBC* White blood cells, *PLTs* Platelets

*P < 0.05 vs. healthy control

Detailed methods are provided in Supplementary materials.

### Statistical analysis

Shapiro–Wilk test was used to test normal distribution of the datasets. Various methods of comparison between two groups were used (Mann-Whitney test) for unpaired samples. It is necessary to use the ordinary one-way ANOVA test, the Brown-Forsythe and Welch’s ANOVA test to make comparisons among more groups. All analyses were performed with Prism 9.0. A *P* value < 0.05 was considered statistically significant.

## Results

### Neutrophils tend to generate more NETs in AIS patients

**Fig. 1:**
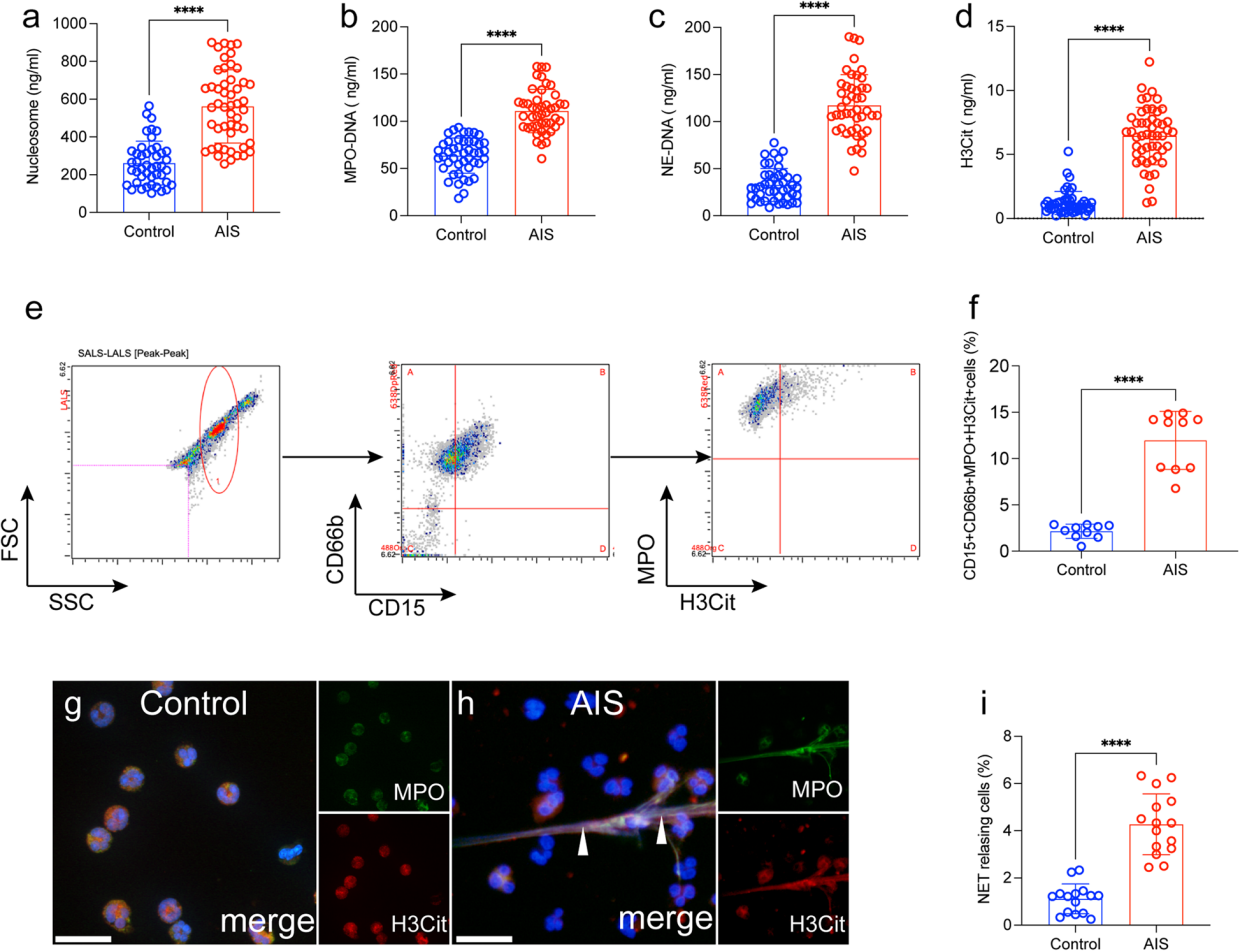
NET markers in stroke patients before and after thrombolysis. NET markers nucleosome (**a**), MPO-DNA (**b**), NE-DNA (**c**) and H3Cit-DNA (**d**) were measured in plasma samples from healthy subjects (*n* = 45) and ischemic stroke patients (*n* = 45) by ELISA. **e** NET releasing cells, defined as CD15 + CD66b + MPO + H3Cit + groups and detected in whole blood samples from healthy subjects (*n* = 10) and AIS patients (*n* = 45). The flow cytometry gate is showed. **f** The rate of NET releasing cells in whole blood samples from each group. Neutrophils from healthy subjects (**g**) and AIS patients (**h**) were stained for MPO (green) and H3Cit (red) and observed by confocal microscopy. **i** The rate of NETosis in neutrophils from each group. The inset scale bars in**e**, **f** and **h**, Iiare 40 μm. Statistics, Mann-Whitney test (**a**-**d**, **f**, **i**). Data are presented as the mean ± SD. ****P < 0.0001

To investigate whether NETs contribute to the development of AIS, nucleosomes, MPO-DNA, NE-DNA, and H3Cit were measured in plasma samples from each group (Fig. 1a-d). The level of NETs in AIS patients were obviously higher compared with healthy controls (Fig. 1a-d). In addition, we detected NET releasing neutrophils (CD15 + CD66b + MPO + H3Cit + cells) in blood samples by flow cytometry. Interestingly, NET releasing neutrophils from AIS patients showed an obvious elevation than those from healthy controls (Fig. 1e and f). Next, we evaluated the potency of NETosis and found that those from AIS patients showed a greater tendency to generate NETs, as observed by Immunofluorescence staining (Fig. 1g-i).

### Platelets microvesicles sustain the formation of NETs by autophagy in AIS patients

**Fig. 2:**
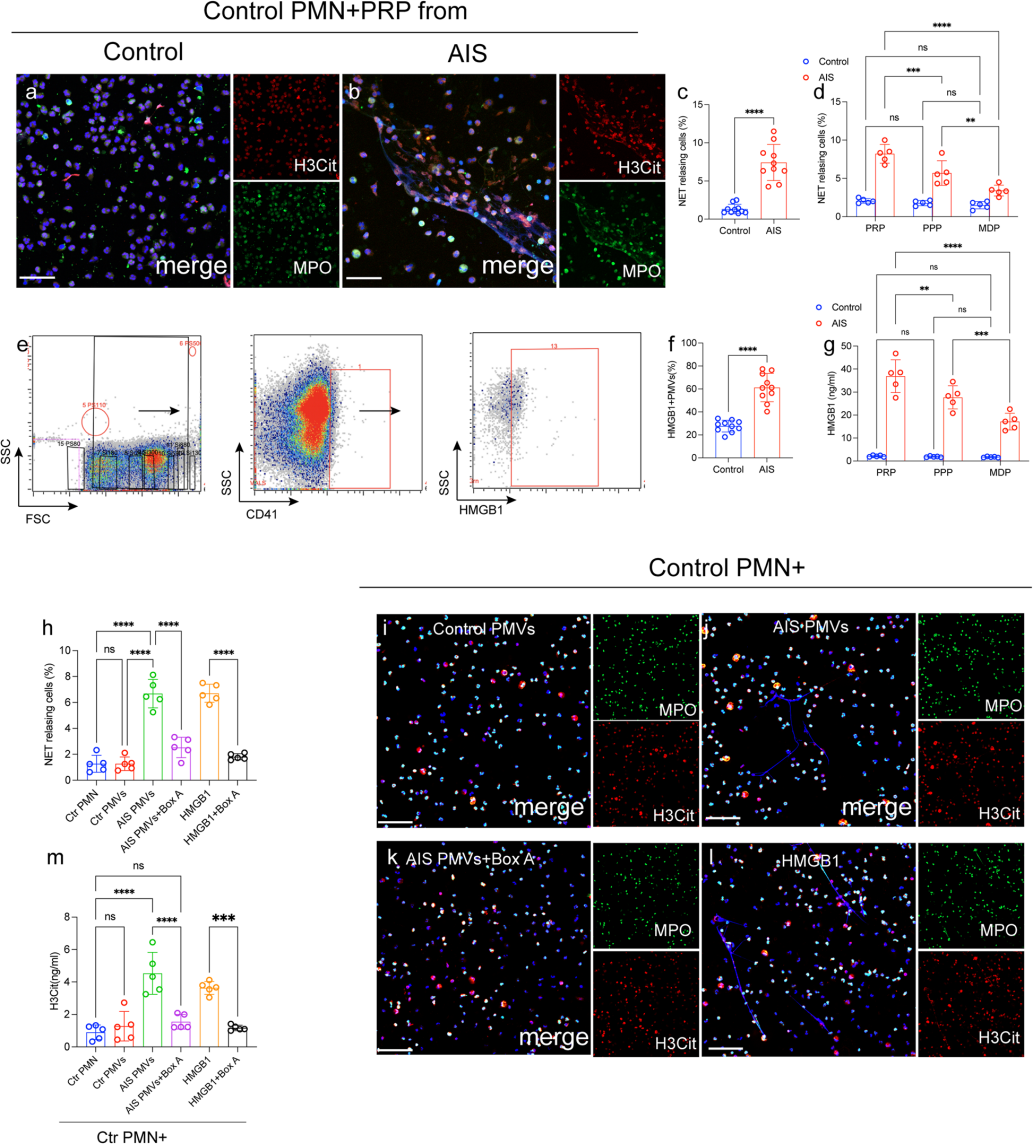
HMGB1 from PMPs induces NET formation. **a**-**c** Neutrophils from healthy individuals were incubated with PRP from AIS patients (*n* = 10) and healthy controls (n = 10). Treated neutrophils were co-stained with MPO (green) and H3Cit (red) and observed by confocal microscopy. **d** NET releasing cells in neutrophils incubated with PPR, PFP and MDP. **e** The flow cytometry gated of HMGB1 + PMPs is showed. **f** HMGB1 + PMPs in samples from healthy subjects (*n* = 10) and AIS patients (*n* = 10) was detected by flow cytometry. Neutrophils were incubated with PMPs in the presence of Box A (the competitive HMGB1 antagonist). The levels of H3Cit in the supernatant from each group was measured by ELISA (**g**) and neutrophils were stained with MPO (green) and H3Cit (red) analyzed by confocal microscopy (**h**-**l**). The inset scale bars in a, b and h-k are 40 μm. Statistics, Mann-Whitney test (**c**, **f**) and ordinary ANOVA (**d**, **g**, **l**). Data are presented as the mean ± SD. ***P < 0.001, and ****P < 0.0001. PMN, polymorphonuclear

Platelets play a vital role in the generation of NETs as previously reported [18, 20, 22]. Hence, we sought to explore the interaction between activated platelets and NETosis in AIS patients. Neutrophils were challenged with PRP from each group. An marked elevation in NET-releasing neutrophils in cells treated with plasma from AIS patients observed by immunofluorescence (Fig. 2a-c). Interestingly, neutrophils incubated with platelet poor plasma (PPP) also could induce NETs formation. However, the rate of NET releasing cells was significantly decrease in neutrophils treated with microparticle depletion plasma (MDP) (Fig. 2d). These findings give us hint that platelet-derived microvesicles (PMPs) may play a crucial role in NET formation in AIS. Then, we investigated the interaction between PMPs and NETs formation in AIS. In whole blood from AIS patients and healthy subjects, PMPs identified by size and CD41 (Fig. 2e and f). PMPs of AIS patients were more likely to express the HMGB1 than microvesicles of healthy controls as assessed by flow cytometry (Fig. 2e and f). Then, we treated control neutrophils with PMPs from each group. As shown in Fig. 2h-k, the higher rate of NET-releasing cells in neutrophils treated with PMPs from AIS patients than those from healthy donners. Next, control neutrophils were incubated with PMPs in the presence of Box A (the competitive HMGB1 antagonist), and confocal images and ELISA results both indicated that Box A significantly blocked the information of NETs mediated by PLT-rich plasma from AIS patients (Fig. 2g).

**Fig. 3:**
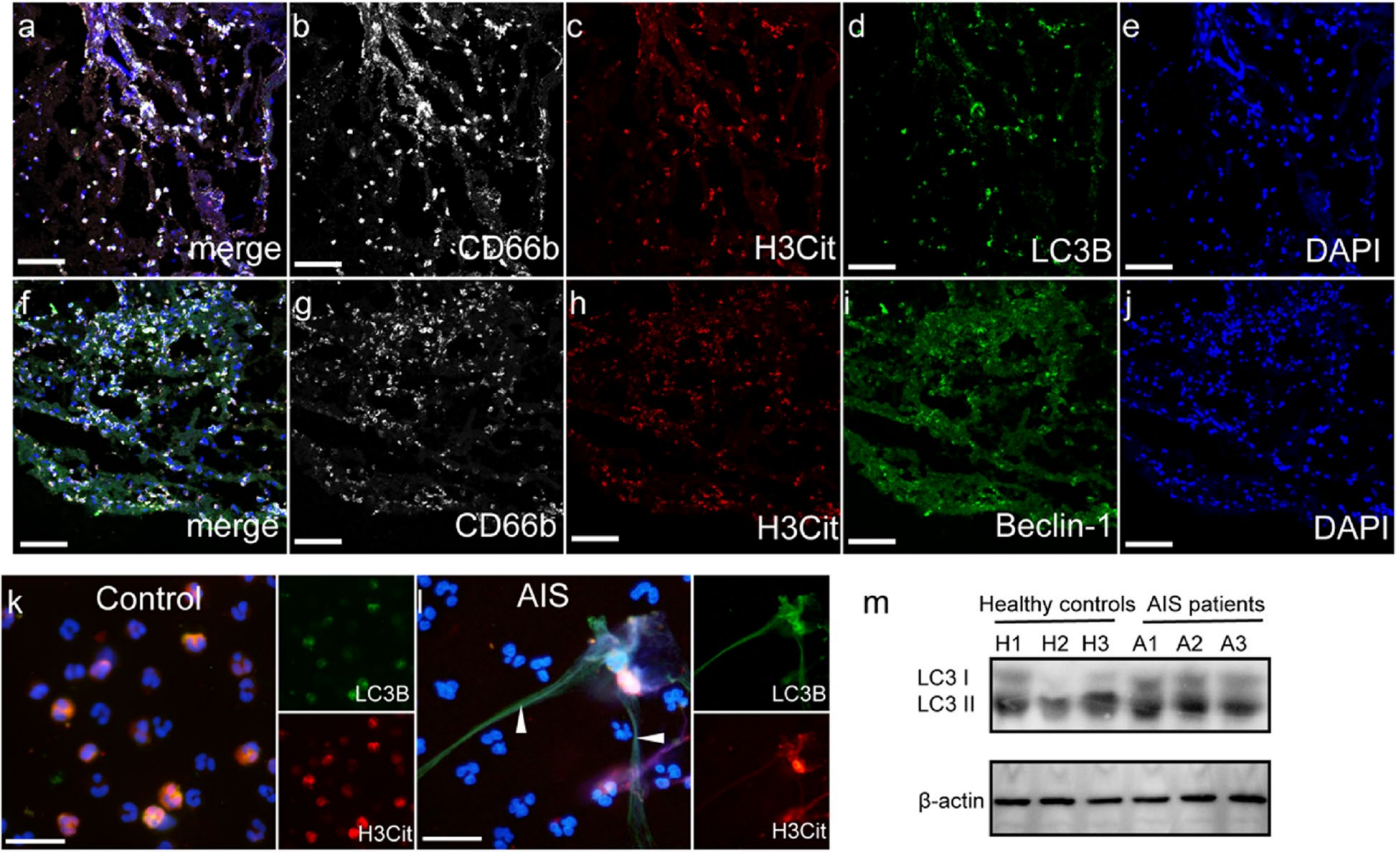
NET markers were colocalized with autophagy associated protein in stroke thrombi and neutrophils. **a**-**j** Stroke thrombi were stained with CD66b (white), H3Cit (red), LC3B (green), Beclin-1 (green) and DAPI (blue). Neutrophils from healthy controls (**k**) and AIS patients (**l**) were stained with H3Cit (red) and LC3B (green) and observed by confocal microscopy. **m** The expression of LC3B on neutrophils from healthy controls (*n* = 3) and AIS patients (*n* = 3). The inset scale bars are 20 μm in **a**-**j**

**Fig. 4:**
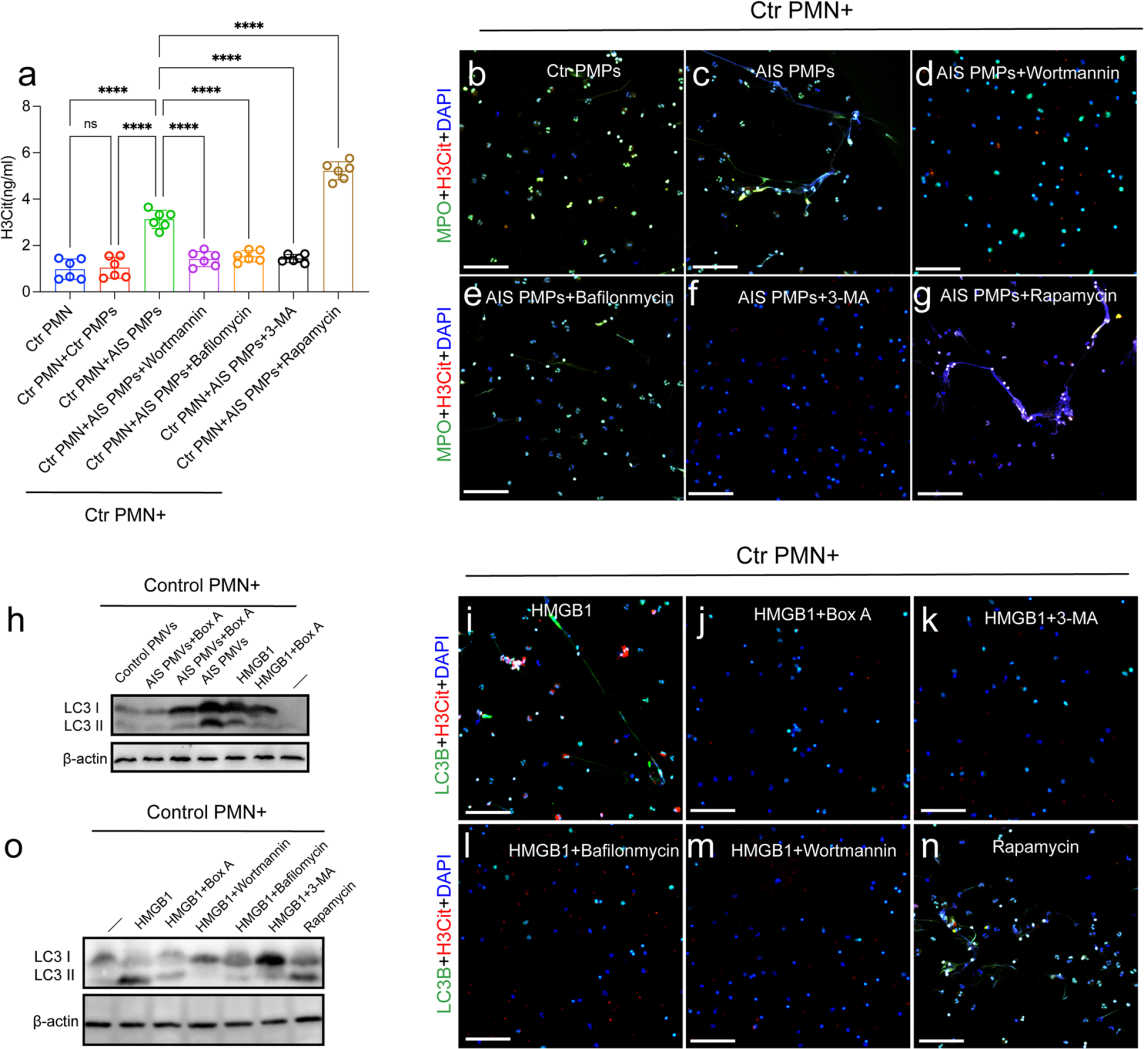
HMGB1 from PMPs promote NETosis through autophagy. Control neutrophils were incubated with PMPs in the presence of with or without wortmannin (150 nM), 3-MA (5 mM), bafilomycin A1 (1µM), rapamycin (100 nM), Box A. The levels of H3Cit in the supernatant from each group was measured by ELISA (**a**) and neutrophils were stained with MPO (green) and LC3B (red) analyzed by confocal microscopy (**b**-**g**). Control neutrophils were incubated with PMPs in the presence of with or without Box A. **h** The expression of LC3B on treated neutrophils were detected by western blotting and neutrophils were stained with MPO (green) and LC3B (red) analyzed by confocal microscopy (**i**-**n**). **o** Control neutrophils were incubated with recombinant HMGB1 in the presence of with or without wortmannin (150 nM), 3-MA (5 mM), bafilomycin A1 (1µM), rapamycin (100 nM) and the expression of LC3B on treated neutrophils were detected by western blotting. The inset scale bars in **b**-**g** and **i**-**n** are 40 μm. Statistics, ordinary one-way ANOVA. Data are presented as the mean ± SD. ****P < 0.0001. PMN, polymorphonuclear

To further explore the definite mechanism of NETosis in AIS, we analyzed stroke thrombi obtained from patients underwent thrombectomy. On confocal images, LC3B and Beclin-1 were strongly expressed in AIS thrombi, which colocalized with NET markers (CD66b and H3Cit (Fig. 3a-j). This interesting finding showed autophagy might play an important role in the formation of NETs, which led us to determine whether these neutrophils expressed autophagy markers. Neutrophils from AIS patients expressed high levels of autophagic proteins in confocal images and western blots (Fig. 4a-c).

To further investigate the role of autophagy in NETosis in AIS, we conducted additional experiments. We then analyze how neutrophil autophagy may contribute to PMP-mediated NETosis and establish a co-incubation system for neutrophils and PMPs. As assessed by ELISA kit, NET formation (H3Cit) was inhibited by autophagy inhibitors and was further induced by rapamycin after coculturing with PMPs from AIS patients (Fig. 4d). Besides, the expression of LC3B on neutrophils incubated with PMPs from the different groups and recombinant HMGB1 protein. Confocal images and western blots showed that PMPs from AIS patients promote the expression of autophagic protein on neutrophils (Fig. 4d-j) and were markedly attenuated in the presence of Box A. To further prove whether autophagy promote NETs generation by HMGB1. Increased rate of NETosis induced by HMGB1 could be significantly decreased by wortmannin, 3-MA, bafilomycin A1, (autophagy inhibitor) (Fig. 4k). These results suggested that HMGB1 from PMPs induces the formation of NETs by autophagy in AIS patients.

### NETs formation with TF expression enhances procoagulant activity in AIS patients

**Fig. 5:**
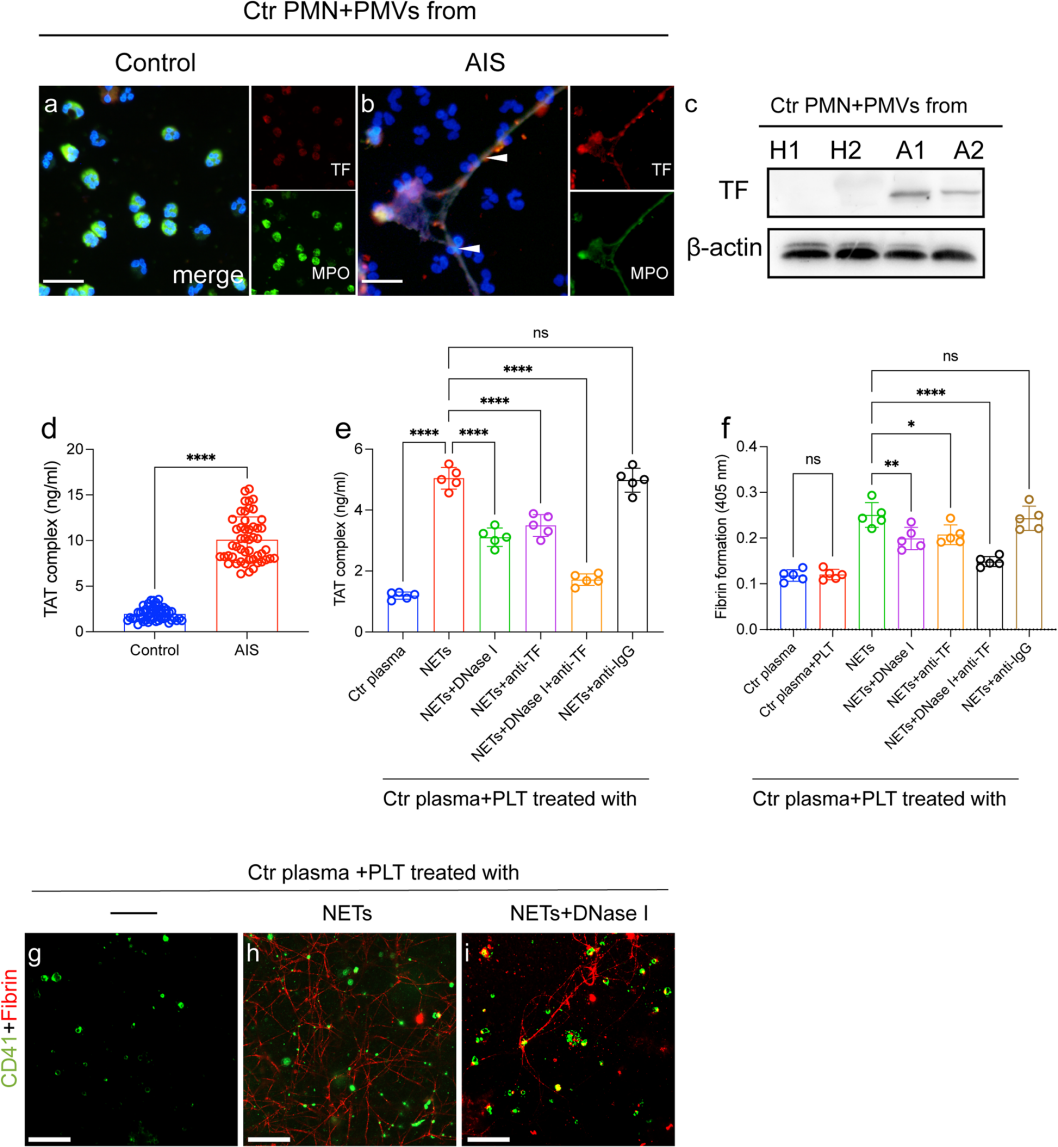
NETs induce procoagulant activity in the AIS process. Control neutrophils were incubated with PMPs from healthy controls (**a**) and AIS patients (**b**) and stained with TF (red) and MPO (green) and observed by confocal microscopy. The expression of TF on neutrophils healthy controls (*n* = 2) and AIS patients (*n* = 2) detected by western blots. **d** Levels of TAT complexes in plasma from healthy subjects (*n* = 45) and AIS patients (*n* = 45) were detected by ELISA. **e** Control plasma was incubated with isolated NETs (0.5 µg DNA/ml) and treated with an anti-TF antibody and DNase I in inhibition assays. **f** Control plasma was incubated PLTs treated with NETs and in the presence of DNase I. **g**-**i** PLTs were stained with CD41 (green) and fibrin (red) in each group. Statistics, Mann-Whitney test (**d**) and ordinary ANOVA (**e**, **f**). Data are presented as the mean ± SD. *P < 0.05, **P < 0.01 and ****P < 0.0001

NETs have been considered as important players in thrombosis [18, 20, 27]. Therefore, we further investigated the NET formation in thrombogenicity in AIS. Neutrophils treated with PMPs from AIS patients tend to expel NET structures decorated with TF expression (Fig. 5a-c).

Based on our results, generation of NETs was accompanied by high TF expression, indicating a potential procoagulant role of NETs in patients receiving thrombolysis treatment. A significant elevation in TAT complex levels was also found in samples from patients with AIS (Fig. 5d). Hence, we evaluated the thrombogenicity of NETs by detecting the TAT complex. Antibodies against DNA traps and TF inhibited DNA trapping and TF binding, respectively, in inhibition assays (Fig. 5e). Degradation of NET-DNA with DNase I decreased thrombin generation by approximately 40%, and combined use of DNase I and an anti-TF antibody decreased thrombin generation by nearly 69%. We then investigated the potential role of NETs in platelet activation. Moreover, fibrin formation of platelets showed a similar trend when treated with NETs, and inhibitors such as DNase I and land anti-TF antibody attenuated the procoagulant effect of platelets (Fig. 5f-i).

### Targeting NETs and PMVs could decrease brain injury and thrombosis in MCAO model

**Fig. 6:**
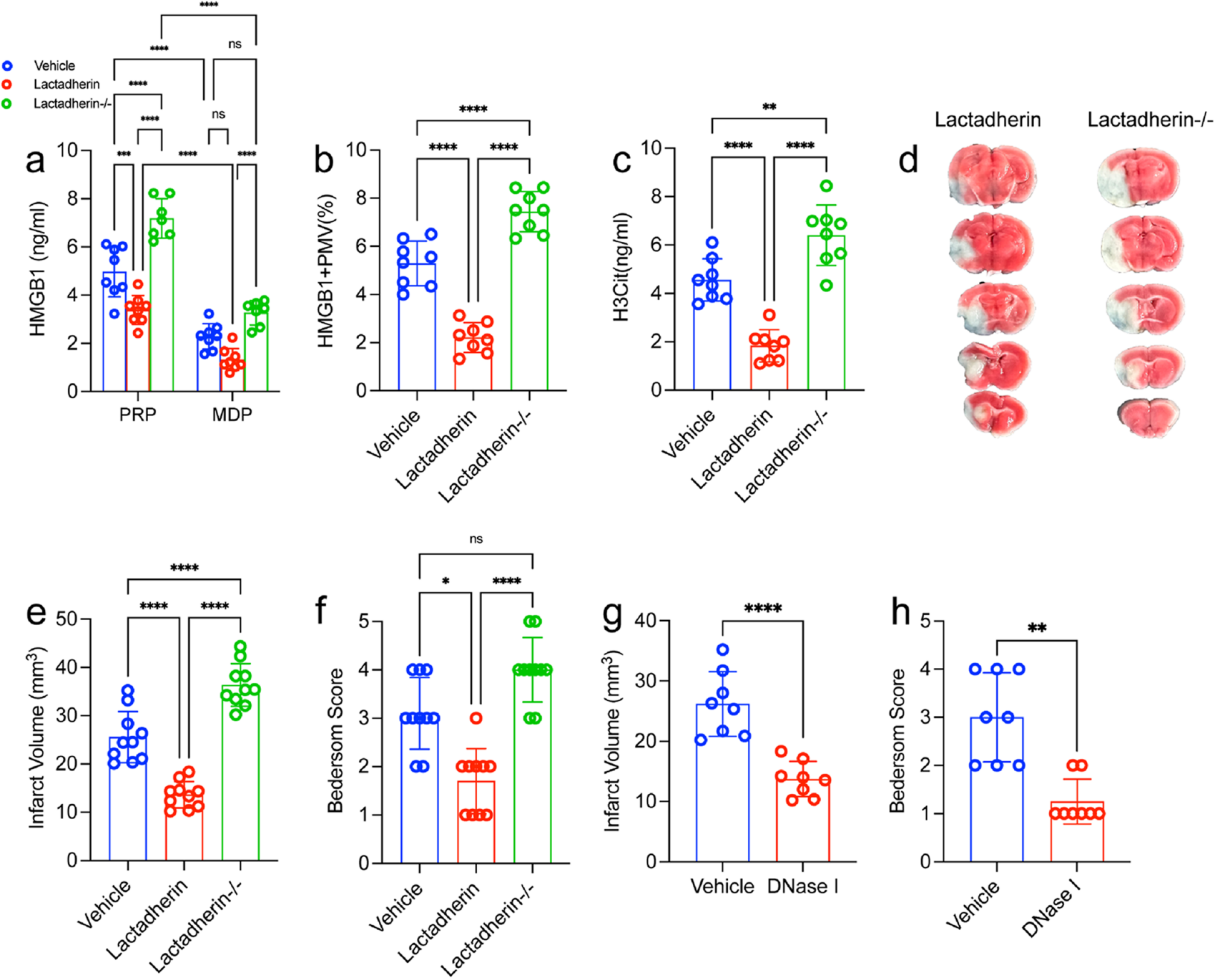
Targeting PMVs could regulate NETs formation and brain injury in MCAO model. **a** PMVs were isolated from lactadherin+/+ or lactadherin-/- mice and incubated for 2 h with neutrophils from WT mice. **b** Plasma HMGB1 levels were measured by ELISA. **c** NET releasing cells from each group. Lactadherin+/+ (WT; *n* = 10) or lactadherin-/-mice or (KO; *n* = 10) mice were subjected to 1 h of transient middle cerebral artery occlusion followed by 23 h of reperfusion. **d** NETs were quantified using an H3Cit ELISA from each group. Plasma was isolated and brains were analyzed for ischemic stroke brain damage by TTC staining, 24 h after stroke onset. **e** Upon TTC staining, live brain tissue will stain red, while dead brain tissue will remain white (outlined with black dotted line). **f** Infarct size was determined by TTC staining and planimetric analysis. **g** Neurological score was measured 24 h after stroke using the Bederson Test. Statistics, Brown-Forsythe and Welch’s ANOVA test (**g**) and ordinary ANOVA (**b**-**d**, **f**). Data are presented as the mean ± SD. *P < 0.05, **P < 0.01, ***P < 0.001 and ****P < 0.0001

To study the interaction HMGB1 drived from PMVs and the formation of NETs in ischemic stroke model, we depleted microvesicles with lactadherin [28] (Fig. 6a). The circulating HMGB1 was significantly lower in mice treated with lactadherin compared to mice treated with control 24 h after stroke. Conversely, inhibiting lactadherin genetically increased the circulating HMGB1 levels (Fig. 6b). Moreover, the plasma HMGB1 was reduced in mice treated with lactadherin and was associated with decreased H3Cit (Fig. 6c and d). Importantly, inhibiting lactadherin could improve the outcomes of ischemic stroke (Fig. 6e-g). In short, our results suggest a critical role for PMPs derived HMGB1 in the formation of detrimental NETs in the acute phase of ischemic stroke.

## Discussion

We obtained four interesting findings. First, circulating NET markers were elevated in AIS patients compared with healthy subjects. Second, HMGB1 was upregulated in PMVs from AIS patients and induced NET formation through autophagy. Third, NETs bound to coagulation factors through highly expressed TF on DNA structures to enhance thrombogenicity. Fourth, PMVs and NETs could be effective therapeutic target for MCAO model.

**Fig. 7:**
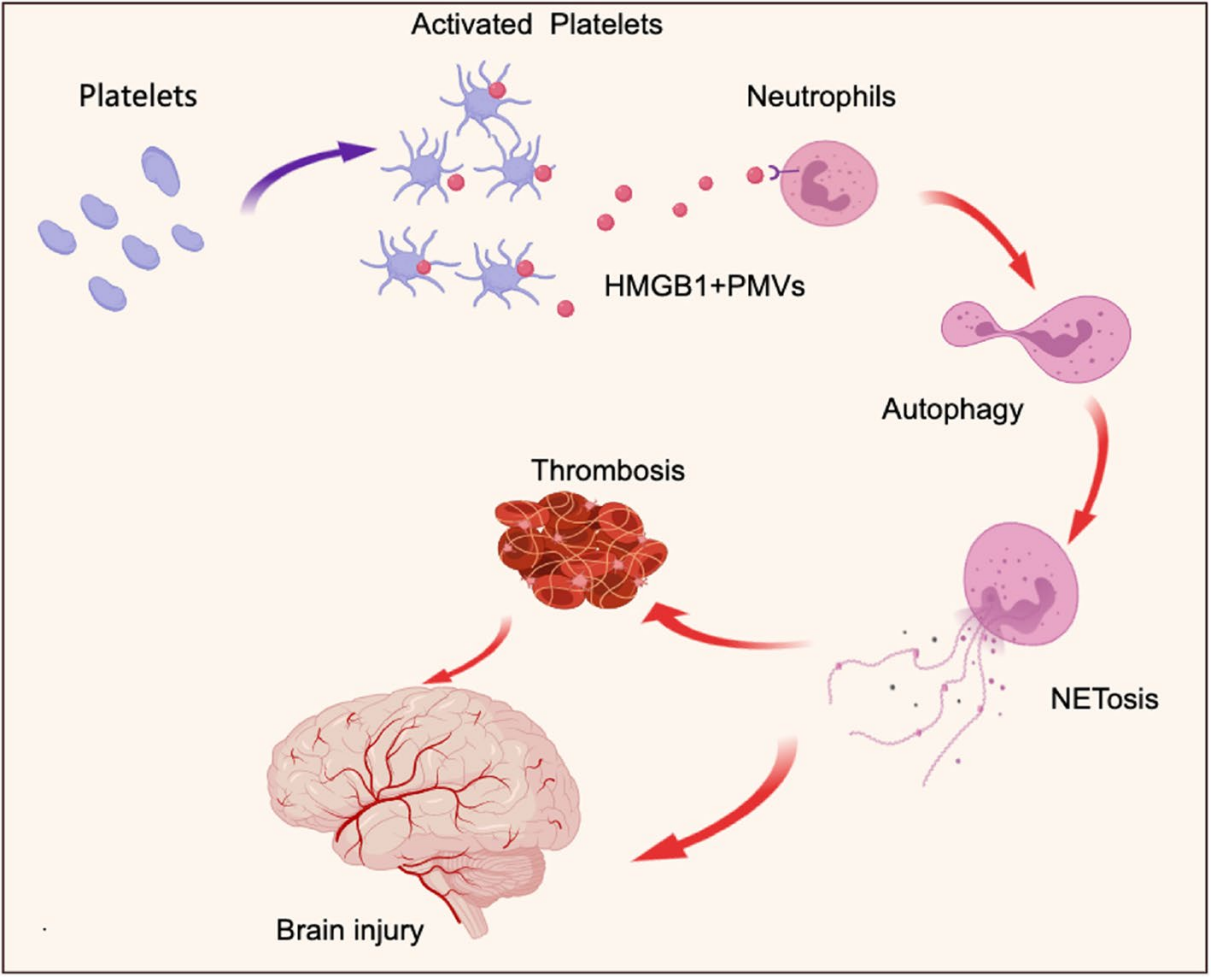
Proposed mechanism of NETs and PMPs contribute to brain injury and thrombosis in AIS. Activated platelets release PMPs in acute phage of AIS. PMPs contain HMGB1 induce neutrophils autophagy associated NETosis and contribute to brain injury and thrombosis in AIS

Neutrophil counts have been studied to be related with poor prognosis in patients with AIS [29, 30]. Moreover, neutrophils are predominantly identified in thrombi from stroke patients in the form of NETs [11–13]. NETs are large and complex DNA-web structures released by activated neutrophils in various diseases [17–19]. Previous studies have explored the potential role of NETs in the acute process of ischemic stroke [20]. In our study, we measured plasma NET levels in samples from AIS patients. Moreover, we also observed that neutrophil counts were positively correlated with the concentration of NET markers in samples from AIS patients. In patients with acute ischemic stroke, there was a significant correlation between total neutrophil counts and infarct volume before 3 days of the symptom onset [31, 32]. The general thrombosis literature has found that activated neutrophils, and particularly NET formation, contribute to the propagation of thrombi in arteries and veins as well as microscopic blood vessels [33, 34]. According to our observation and previous studies, early intervention of NETs may be an important measure to improve the prognosis of patients. Our previous study detected an interaction between NETs and platelets in acute stroke patients with ICA occlusion [20]. Platelets have been considered to act as critical initiators of NET formation in some diseases, such as sepsis [22], myocardial infarction [17] and heparin-induced thrombocytopenia [27]. Building on our previous study, we investigated the potential mechanisms of platelet-NET interactions in the AIS process and found PLT-rich plasma collected from AIS patients to be critical for NET formation in the AIS process. Moreover, neutrophils treated with MDP showed an obvious reduction in NETosis than those treated with PRP from AIS patients. These findings suggested PMVs may have a potential role of NETs formation in AIS. Flow cytometry results showed the high rate of HMGB1 + PMVs in samples from AIS patients. HMGB1 + PMVs have been reported to induce NETs generation in previous studies [35, 36]. We also observed that NET markers were co-localized with autophagy markers in thrombi sections. Neutrophil autophagy has been considered as a pivotal mechanism for NET formation in various studies [37–46]. In our present studies, in vitro co-culture studies revealed that PMVs mediate the NET formation through autophagy in AIS. In a recent study, HMGB1 from platelets mediated NETosis contribute to brain injury in ischemic stroke [47]. Beyond this study, we found PMVs was the important carrier of HMGB1 in AIS based on our present study. As a cellular adhesion protein, lactadherin is responsible for neovascularization and for clearing apoptotic cells [28, 48]. Moreover, lactadherin could clear PMVs in vivo. In our studies, lactadherin could decrease plasma HMGB1 and H3Cit (NET marker) in tMCAO model. Conversely, inhibiting lactadherin in genetical could increase the HMGB1 and H3Cit, indicating the role of PMVs on NETosis in AIS. Our findings revealed that combing targeting PMVs by lactadherin may be a safe and effective way to prevent brain injury in AIS (Fig. 7).

Although thrombolysis has been the major therapy for AIS in recent years, the ability to dissolve thrombi using t-PA is relatively limited. In fact, in a previous study, t-PA resistance occurred in 50% of all stroke patients [49–51]. The mechanism underlying t-PA resistance is complicated and poorly understood. NETs have been identified as an important component of thrombi from stroke patients and may be a potential therapeutic target for t-PA resistance [11–13]. Our results showed persistently high concentrations of NETs in the plasma of AIS patients. Furthermore, we evaluated the TAT complex and fibrin formation in NETs from patients, and our results revealed that NETs from AIS patients contribute to procoagulant activity. We also found that NETs enhance the hypercoagulation state by activating platelets. Thrombolytic drugs have been thought to directly act on the site of thrombolysis to maximize activation of plasminogen and effectively dissolve the thrombus. Nonetheless, we found in our present study that circulating NETs contribute to a hypercoagulation state, which may result in t-PA resistance.

It is important to note, however, that our study has some limitations. First, we analyzed data from a relatively small group of patients at a local hospital. Therefore, future studies with larger sample sizes should be conducted to confirm our conclusions. Second, our present study showed that HMGB1 from PMVs is one of the contributing factors for NET generation in the acute phase of AIS. However, dying cells, such as neurons, can secrete a large amount of HMGB1 into the circulation. Our future work will concentrate on the in-depth mechanistic link between HMGB1 and NETs in AIS. Third, our co-incubation assays revealed that the PMV/neutrophil ratios and HMGB1 concentrations, although consistent with previous findings, exhibited elevated levels in comparison to those observed in patient plasma. To enhance the credibility of our experimental outcomes, we intend to enhance our methodology in future investigations. Furthermore, while lactadherin has been considered efficacious in facilitating the clearance of MVs, it does not exhibit specificity towards platelet-derived MVs. Consequently, future research endeavors should focus on identifying novel targeted interventions for the clearance of diverse types of MVs.

In conclusion, we showed that NETs are involved in the thrombosis and brain injury in AIS. Moreover, we demonstrated that HMGB1 is upregulated in PMPs from patients and induce NET formation. Additionally, we found that targeting PMPs by lactadherin could improve the brain injury in MCAO model. These results may provide new targets for preventing and combating vascular complications in stroke patients.

## Supplementary Information

**Additional file 1.**

## Data Availability

The data required to reproduce these findings cannot be shared at this time due to technical or time limitations. Data are available from the corresponding author upon reasonable request after article published.
