# Supplementary material for: Neutrophil extracellular traps mediated by platelet microvesicles promote thrombosis and brain injury in acute ischemic stroke
Source: Cell Commun Signal. 2024 Jan 17;22:50. doi: 10.1186/s12964-023-01379-8 (PMC10795390; doi:10.1186/s12964-023-01379-8)

**Supplementary methods**

**Human samples**

Citrated-anticoagulated venous blood was obtained from patients before treatment (thrombolysis and thrombectomy) within the first 24 h after the onset of the acute event and from the healthy subjects after a night fast into siliconized glass tubes. Blood from healthy subjects and patients was obtained with informed consent. According to the manufacturer's instructions, human neutrophil separation medium (TBD) was used to isolate human peripheral blood neutrophils. Briefly, a pipette was used to carefully absorb the blood sample, and the sample was added to the liquid surface of the separation solution and centrifuged for 20-30 min at 500-550 g. A pipette was used to carefully absorb the neutrophil layer in the separation solution. Following centrifugation at 150 x g for 15 minutes at room temperature, platelet-rich plasma (PRP) was isolated and used for experiments right away. PLT isolation was performed as previously described[1]. PMVs and microvesicles depleted plasma (MDP) were obtained as previous described[2]. 5 ml Na-citrate tube for PLTs, PRP, and PFP isolation or to an ethylenediaminetetraacetic acid (EDTA) tube for neutrophil isolation.

**NET quantification**

Quantification of myeloperoxidase (MPO)-DNA, neutrophil elastase (NE)-DNA performed by using modified enzyme-linked immunosorbent assays (ELISAs) as previously described[3-5]. Citrullinated histone H3 (H3Cit) and nucleosome was quantified by human H3Cit ELISA kit (JingmeiBiotechnology) and human nucleosome ELISA kit (Jingkangbiotechnolgy).

**NET isolation**

Neutrophils from healthy subjects were treated with 500 nM PMA for 4 h. After removal of the supernatant, NETs adhered at the bottom were washed down by pipetting 2 ml of cold PBS and were centrifuged at for 10 min at 450 x g at 4 °C. Divide supernatant into 1.5 ml micro-centrifuge tubes and spin for 10 min at 18,000 x g at 4 °C. The cell-free supernatant containing NETs (DNA–protein complex) was collected. The DNA concentration of NETs was measured by spectrophotometry and the isolated NETs were used for further experiments[6, 7].

**Platelet microvesicles (PMVs) identification and isolation**

The microvesicles-gate was calibrated using Apogee mix-sized calibration beads (Cat# 1527, Apogee Flow Systems, Hertforshire), which contain a mixture of plastic spheres (80, 110, 180, 240, 300, 500, 590, 880, 1300 nm). Microvesicles were identified by forward scatter (size) and side scatter (complexity) and defined as particles *<*880 nm in size (threshold based on forward scatter) as previously described[8].

PMVs isolation was performed as previously described[9-11]. Samples containing 5ml of washed platelets rom healthy subject or AIS patients were stimulated for 30 min at 37 °C under constant stirring with 0.1 U/ml of thrombin. Platelets were pelleted by low-speed centrifugation (750 x *g*, 20 min) and the supernatant was then centrifuged at 20,000 x g for 90min at 10 °C to collect PEVs. PEVs were resuspended in 25mM Tris/HCl, 0.1% SDS, pH 7.4 for proteomic analysis and in HEPES buffer for all the other experiments.

**In vitro stimulations and inhibition assays**

For in vitro studies, to test potency of neutrophils in NETs formation spontaneously, PMNs were isolated from peripheral blood from healthy subjects and AIS patients and were cultured in 5% CO_2_ at 37℃in RPMI (Gibco) for 4h (Figure 1g-i). Control neutrophils were also incubated with PRP (20%) derived from citrate blood of healthy subjects and AIS patients for 4 hours (Figure 2a-c).

To explore the possible involvement of PMVs in the development of NETs, the following PMVs-neutrophil co-culture system was developed. Neutrophils from healthy subjects were seeded onto 24-well plates at 1 x 10^5^ cells per well and incubated for 1 h with PMVs (microparticle:neutrophil ratio = 20:1) from each group in the presence of with or without wortmannin (150 nM, MedChemExpress), 3-MA (5 mM, MedChemExpress), bafilomycin A1 (1μM, MedChemExpress), rapamycin (100 nM, MedChemExpress) (Figure 3a-g, Figure5 a-c).

To determine if HMGB1 induced NET formation through autophagy, Neutrophils from healthy subjects were seeded onto 24-well plates at 1 x 10^5^ cells per well and incubated for 1 h with PMVs (microparticle:neutrophil ratio = 20:1) from each group in the presence of recombinant HMGB1 (HMG Biotech, 10 μg/mL) and Box A (HMG Biotech, 10 μg/mL), wortmannin (150 nM, MedChemExpress), 3-MA (5 mM, MedChemExpress), bafilomycin A1 (1μM, MedChemExpress), rapamycin (100 nM, MedChemExpress) (Figure 3i-o).

**Platelet stimulation assays**

Washed platelets (0.5 x 10^8^ PLT/ml) were seeded on slides blocked with 5% BSA in PBS[12]. The platelets were incubated for 2 h with isolated NETs (0.5 μg DNA/ml) in the presence of DNase I (100 U/mL, Roche). For fibrin formation, 100 μl platelets (0.5x10^8^ PLT/ml) was incubated with 50 μl platelet-poor plasma from healthy subjects for 2 min, followed by the addition of 50 μl of prewarmed 25 mmol/l CaCl_2_ for 2 h.

**TAT-complex assays**

Isolated NETs (0.5 μg DNA/ml) were incubated with 50 μl of platelet-poor plasma from healthy individuals for 2 min, followed by the addition of 50 μl of prewarmed 25 mmol/l CaCl_2_ for 2 h. Levels of the TAT complex were detected using a TAT complex ELISA kit (Jingkang, Shanghai) according to the manufacturer’s protocol.

**Flow cytometry**

To investigate NET releasing cells in whole blood samples from each group, one hundred microliters of citrated blood freshly collected from healthy subjects and patients was diluted in PBS and stained with anti-CD15 (APC-Cy7, Bioledgend 323048), anti-CD66b (PE, Bioledgend 303720), Alexa Fluor 647-conjugated H3Cit and anti-MPO (FITC, Bioledgend 347201). Alexa Fluor 647-conjugated H3Cit was conducted with Alexa Fluor 647 Conjugation Kit (Fast) - Lightning-Link (ab269823) and anti-histone H3 antibody (citrulline R8, ab232939), in accordance with the protocol contained in the publication. To investigate HMGB1 on PMVs in whole blood samples from each group, one hundred microliters of citrated blood freshly collected from healthy donners and AIS patients and incubated with anti-CD41 (Alexa Fluor 488, Biolegend 303724), anti-HMGB1 (PE, Biolegend 303720). Analyzing Microparticles (MP) were performed by APogeeMix (Cat#1527) and investigated by flow cytometry (Apogee A60 flow cytometer).

**Confocal imaging**

Neutrophils were stained with anti-histone H3 citrulline, anti-MPO and anti-tissue factor (TF, ab228968) antibodies. PLTs were stained with anti-CD41(Novus) and anti-fibrin (Novus) antibodies. The frozen tissue samples were cut into 8 μm sections and stained anti-histone H3, anti-CD66b (Affinifty), anti-Beclin-1(Proteintech), and anti-LC3B (CST, 83506) antibodies. All immunofluorescence imaging was analyzed by confocal microscopy (Zeiss, LSM 880).

**Animal studies**

C57Bl/6J mice (12-16 weeks old and 22-25 g) were purchased from the Animal Laboratory Center of the Second Affiliated Hospital of Harbin Medical University. Lactadherin-deficient (lactadherin-/-) mice on a C57Bl/6J (12-16 weeks old and 22-25 g) background were purchased from Cyagen Biosciences. Transient occlusion of the right middle cerebral artery (MCAO) is performed as previously described[13]. Thirty minutes before MCAO, the mice received a single tail vein infusion of either 400 ug/kg (;10 mg/mouse) purified lactadherin. The mouse study was approved by the Institutional Animal Care and Use Committee of the Ethics Committee of The Second Affiliated Hospital of Harbin Medical University. To quantify ischemic stroke brain damage, 2-mm-thick coronal brain sections were stained with 2% 2,3,5-triphenyl-tetrazolium chloride (TTC, Solarbiot) to distinguish unaffected brain tissue from infarcted tissue, 24 hours after stroke induction. Stained slices were photographed and infarct areas (white) were measured using Image J software by an operator blinded for treatment.

**References**

1. Koupenova M, Corkrey HA, Vitseva O, Manni G, Pang CJ, Clancy L, Yao C, Rade J, Levy D, Wang JP, et al: **The role of platelets in mediating a response to human influenza infection.** *Nat Commun* 2019, **10:**1780.

2. Novak M, Hiden M, Rehak T, Rosenkranz A, Zebisch A, Sill H, Klaschka S, Muntean W: **Enhanced thrombin generation in plasma of severe thrombocytopenic patients due to rFVIIa.** *Hamostaseologie* 2008, **28 Suppl 1:**S77-80.

3. Jiao Y, Li W, Wang W, Tong X, Xia R, Fan J, Du J, Zhang C, Shi X: **Platelet-derived exosomes promote neutrophil extracellular trap formation during septic shock.** *Crit Care* 2020, **24:**380.

4. Zhou P, Li T, Jin J, Liu Y, Li B, Sun Q, Tian J, Zhao H, Liu Z, Ma S, et al: **Interactions between neutrophil extracellular traps and activated platelets enhance procoagulant activity in acute stroke patients with ICA occlusion.** *EBioMedicine* 2020, **53:**102671.

5. Jin J, Zhao X, Li W, Wang F, Tian J, Wang N, Gao X, Zhang J, Wu J, Mang G, et al: **Neutrophil extracellular traps: A novel therapeutic target for intracranial hemorrhage.** *Thromb Res* 2022, **219:**1-13.

6. Najmeh S, Cools-Lartigue J, Giannias B, Spicer J, Ferri LE: **Simplified Human Neutrophil Extracellular Traps (NETs) Isolation and Handling.** *J Vis Exp* 2015.

7. Yang L, Liu Q, Zhang X, Liu X, Zhou B, Chen J, Huang D, Li J, Li H, Chen F, et al: **DNA of neutrophil extracellular traps promotes cancer metastasis via CCDC25.** *Nature* 2020, **583:**133-138.

8. Zhao X, Han J, Zhou L, Zhao J, Huang M, Wang Y, Kou J, Kou Y, Jin J: **High mobility group box 1 derived mainly from platelet microparticles exacerbates microvascular obstruction in no reflow.** *Thromb Res* 2023, **222:**49-62.

9. Vismara M, Manfredi M, Zara M, Trivigno SMG, Galgano L, Barbieri SS, Canobbio I, Torti M, Guidetti GF: **Proteomic and functional profiling of platelet-derived extracellular vesicles released under physiological or tumor-associated conditions.** *Cell Death Discov* 2022, **8:**467.

10. Vismara M, Zara M, Negri S, Canino J, Canobbio I, Barbieri SS, Moccia F, Torti M, Guidetti GF: **Platelet-derived extracellular vesicles regulate cell cycle progression and cell migration in breast cancer cells.** *Biochim Biophys Acta Mol Cell Res* 2021, **1868:**118886.

11. Zara M, Guidetti GF, Boselli D, Villa C, Canobbio I, Seppi C, Visconte C, Canino J, Torti M: **Release of Prometastatic Platelet-Derived Microparticles Induced by Breast Cancer Cells: A Novel Positive Feedback Mechanism for Metastasis.** *TH Open* 2017, **1:**e155-e163.

12. Morrow GB, Whyte CS, Mutch NJ: **Functional plasminogen activator inhibitor 1 is retained on the activated platelet membrane following platelet activation.** *Haematologica* 2020, **105:**2824-2833.

13. South K, Saleh O, Lemarchand E, Coutts G, Smith CJ, Schiessl I, Allan SM: **Robust thrombolytic and anti-inflammatory action of a constitutively active ADAMTS13 variant in murine stroke models.** *Blood* 2022, **139:**1575-1587.

**Supplemental Figure 1**

Neutrophil counts were positively correlated with MPO-DNA (A), NE-DNA (B), Nucleosome (C) and H3Cit (D) in plasma from AIS patients (n=50). Statistical analysis was performed with Spearman correlation test.


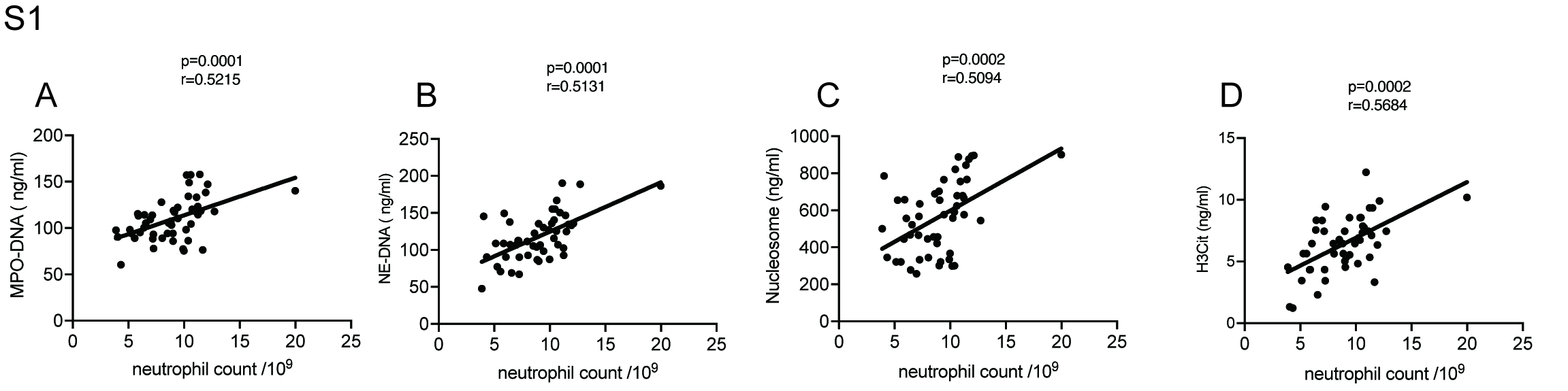


**Supplemental Figure 2**

To evaluate the interaction NETs between HMGB1+MVs, we established tMCAO model. In our results, we found the rate of HMGB1+PMVs were significantly decreased in DNase I group (n=5) than those from Vehicle group (n=5). Statistical analysis was performed with t-test.


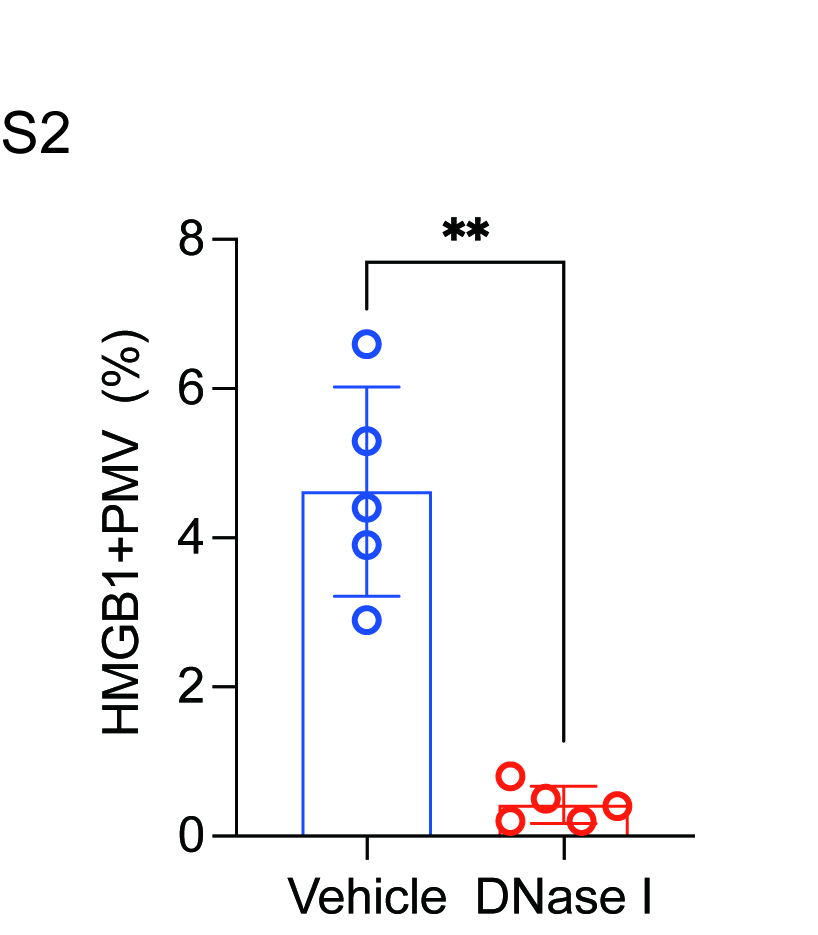

Supplement: Supplementary file 2 — Additional file 1. [file 12964_2023_1379_MOESM1_ESM.zip › AIS-Supplementary methods-R2.docx]
