# Supplementary figures and images for: Neutrophil extracellular traps mediated by platelet microvesicles promote thrombosis and brain injury in acute ischemic stroke
Source: Cell Commun Signal. 2024 Jan 17;22:50. doi: 10.1186/s12964-023-01379-8 (PMC10795390; doi:10.1186/s12964-023-01379-8)

Figure 3m

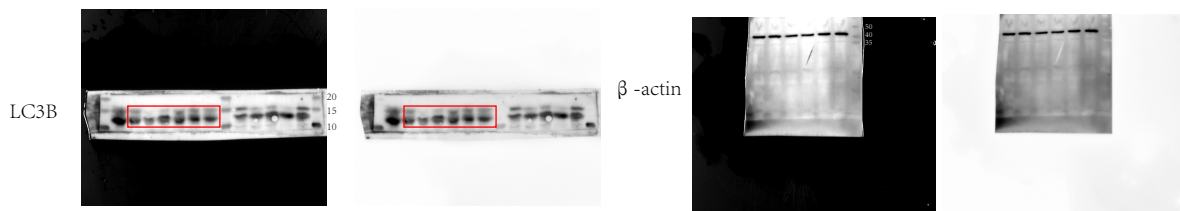

Figure 4 h

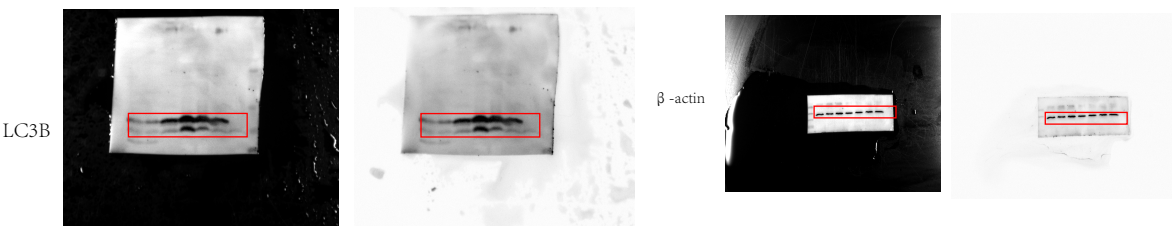

Figure 4o

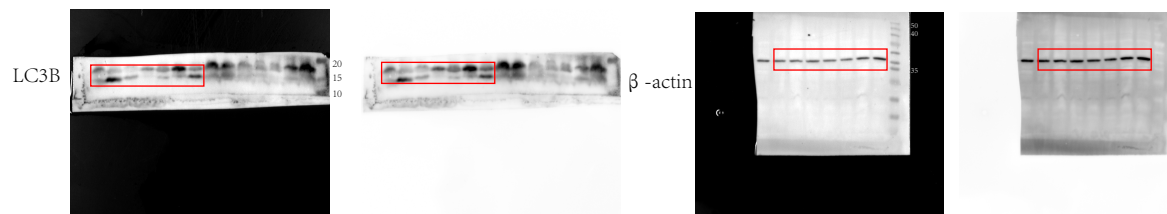

Figure 5C

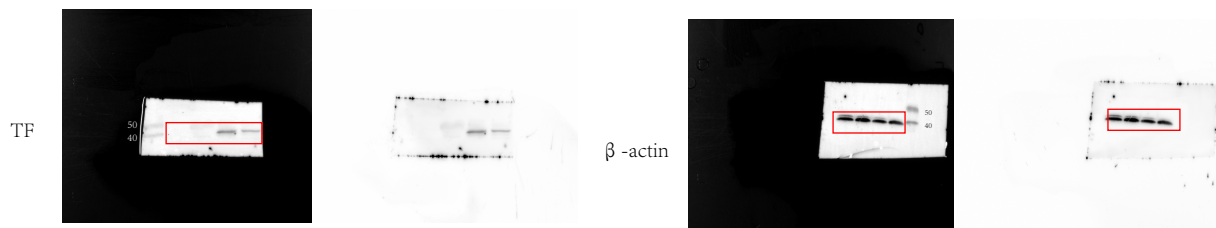

Supplement: Supplementary file 2 — Additional file 1. [file 12964_2023_1379_MOESM1_ESM.zip › WB original blot-R.pdf]
